# Developmental, ultrastructural and cytochemical investigations of the female gametophyte in *Sedum rupestre* L. (Crassulaceae)

**DOI:** 10.1007/s00709-020-01584-z

**Published:** 2020-11-14

**Authors:** Emilia Brzezicka, Małgorzata Kozieradzka-Kiszkurno

**Affiliations:** Department of Plant Cytology and Embryology, Faculty of Biology, University of Gdańsk, 59 Wita Stwosza St., 80-308 Gdańsk, Poland

**Keywords:** Antipodal cells, Cytochemistry, Embryo sac, Megagametogenesis, Megasporogenesis, Ultrastructure

## Abstract

This article describes the development of female gametophyte in *Sedum rupestre* L. New embryological information about the processes of megasporogenesis and megagametogenesis provided in this paper expand the current knowledge about the embryology of the studied species. *S*. *rupestre* is characterized by monosporic megasporogenesis and the formation of *Polygonum*–type embryo sac*.* The process of megasporogenesis is initiated by one megaspore mother cell, resulting in the formation of a triad of cells after meiosis and cytokinesis. The functional megaspore, which is located chalazally, is a mononuclear cell present next to the megaspore in the centre of the triad. Only one of the two non-functional cells of the triad is binucleate, which occur at the micropylar pole. In this paper, we explain the functional ultrastructure of the female gametophytic cells in *S. rupestre*. Initially, the cytoplasm of the gametophytic cells does not differ from each other; however, during differentiation, the cells reveal different morphologies. The antipodals and the synergids gradually become organelle-rich and metabolically active. The antipodal cells participate in the absorption and transport of nutrients from the nucellar cells towards the megagametophyte. Their ultrastructure shows the presence of plasmodesmata with electron-dense material, which is characteristic of Crassulaceae, and wall ingrowths in the outer walls. The ultrastructure of synergid cells is characterized by the presence of filiform apparatus and cytoplasm with active dictyosomes, abundant profiles of endoplasmic reticulum and numerous vesicles, which agrees with their main function—the secretion of pollen tube attractants. Reported data can be used to resolve the current taxonomic problems within the genus *Sedum* ser. *Rupestria*.

## Introduction

Carpels and stamens are two flower parts responsible for the process of sexual reproduction in angiosperms. These reproductive structures are the production sites of female (embryo sac) and male (pollen grain) gametophytes, respectively. An ovule is an organ that develops in the basal region of carpels called the ovary. It is made of diploid sporophytic cells, including nucellar tissue in which the gametophytic cells undergo differentiation (Raghavan 2000; Bhojwani et al. 2015). In most of the flowering plants, one uninucleate cell, resulting from meiosis (monosporic megasporogenesis), initiates the formation of megagametophyte (megagametogenesis). This functional megaspore (FM), which is mostly located at the chalazal pole, is the only surviving and developing cell that becomes an embryo sac. The formation of a seven-celled female gametophyte of *Polygonum* type from one chalazally located megaspore has been observed in a majority (over 70%) of angiosperms (Johri et al. 2001; Yadegari and Drews 2004; Drews and Koltunow 2011).

Furthermore, the formation of a *Polygonum*-type female gametophyte is described as most common among the Crassulaceae species investigated so far (Mauritzon 1933; Thiede and Eggli 2007). Embryological data have been published for some Crassulaceae members, but these are limited and insufficient in the light of microscopical observations, inter alia, made during the development of female gametophyte. Moreover, illustrations constitute the major part of the documents that present the female gametophyte development of some representative members of this family. This is due to the fact that light microscopy has been used predominantly for the analysis of Crassulacean ovules at the stages of megasporogenesis and megagametogenesis (Rombach 1911; Sharp 1913; Souéges 1927; Mauritzon 1933; Johri et al. 1992; Wojciechowicz and Samardakiewicz 1998; Thiede and Eggli 2007). Until now, cytochemical and ultrastructural analyses have been carried out mainly during embryogenesis in some Crassulaceae members and the following genera—*Sedum* (*S. acre* L., *S. hispanicum* L., *S. sediforme* (Jacq.) Pau, *S. reflexum* L., *S. album* L., *S. atratum* L.), *Sempervivum* (*S. arachnoideum* L.), *Jovibarba* (*J. sobolifera* (Sims) Opiz), *Graptopetalum* (*G. bellum* L.), *Aeonium* (*A*. *sedifolium* (Webb ex Bolle) Pit. & Proust), *Monanthes* (*M*. *anagensis* Praeger), *Aichryson* (*A*. *laxum* (Haw.) Bramwell) and *Echeveria* (*E*. *lutea* Rose) (Kozieradzka-Kiszkurno and Bohdanowicz 2006, 2010; Kozieradzka-Kiszkurno et al. 2011, 2012, 2020; Kozieradzka-Kiszkurno and Płachno 2012; Czaplejewicz and Kozieradzka-Kiszkurno 2013; Majcher 2017). To the best of our knowledge, cytochemical and ultrastructural analyses during the formation of megaspores and megagametophyte have been conducted on ovules collected from only two *Sedum* species—*S. hispanicum* L. and *S. sediforme* (Jacq.) Pau (Brzezicka and Kozieradzka-Kiszkurno 2018, 2019).

Among the angiosperms, the family Crassulaceae has been described with new, different structural features in embryological studies. Except for *S. reflexum*, these distinct structural observations, in the form of plasmodesmata covered by an electron-dense material, were noted for all the above-listed Crassulaceae species during embryogenesis and/or megasporogenesis and/or megagametogenesis. Interestingly, plasmodesmata with unusual electron-dense material have been not observed during embryogenesis in two species from genus *Sedum* ser. *Rupestria* (*S. sediforme* and *S. reflexum*) which were ultrastructurally tested thus far (Czaplejewicz and Kozieradzka-Kiszkurno 2013; Majcher 2017). However, for *S. sediforme*, it was confirmed that the absence of plasmodesmata with electron-dense dome during embryogenesis does not exclude the possibility of their formation during the development of female gametophyte (Brzezicka and Kozieradzka-Kiszkurno 2019). This has not been proven for *S. reflexum*. Therefore, in our study, we test the hypothesis that *Sedum rupestre* L. (syn. *S*. *reflexum* L. following Gallo and Zika 2014 and The Plant List 2013), as a species closely related species to *S*. *sediforme*, shows the presence of plasmodesmata with an electron-dense material in the cell walls of developing female gametophyte.

Approximately 420 species belong to the largest genus *Sedum* within the Crassulaceae family (Christenhusz and Byng 2016)*.* However, it is known that *Sedum* is a highly polyphyletic group, and hence, has been an object of interest for taxonomists. By contrast, the genus ser. *Rupestria* Berger is a monophyletic group, raised repeatedly to the other, new genus—*Petrosedum* Grulich (Thiede and Eggli 2007, Gallo and Zika 2014 and literature therein). The morphological and embryological features of these plants are simultaneously an argument, which favour their classification. As indicated by Thiede and Eggli (2007), the morphology of the described species is different from that of other Euro-Mediterranean *Sedum.* Some researchers have conducted ultrastructural and cytochemical analyses on the ovules of ser. *Rupestria* at the stages of embryogenesis (*S. reflexum* L. and *S. sediforme*) (Czaplejewicz and Kozieradzka-Kiszkurno 2013; Majcher 2017; Kozieradzka-Kiszkurno et al. 2020), megasporogenesis and megagametogenesis (*S. sediforme*) (Brzezicka and Kozieradzka-Kiszkurno 2019). Mauritzon (1933) carried out embryological studies on two species of ser. *Rupestria*—*S. rupestre* L. and *S. anopetalum* (the name is a synonym of *S. ochroleucum* Chaix following The Plant List 2013) using light microscopy. However, the author described the megasporogenesis process and the structure of the embryo sac only for *S. anopetalum*. It can be summarized that in all the mentioned species of ser. *Rupestria*, the suspensor is filamentous (composed of one basal cell and chalazal cells arranged in a row) and morphologically different from that described for most *Sedum* and Crassulaceae species. For instance, in *S. reflexum* and *S. sediforme*, the suspensors are made of ten cells (Mauritzon 1933; Czaplejewicz and Kozieradzka-Kiszkurno 2013; Majcher 2017; Kozieradzka-Kiszkurno et al. 2020). The proposition to recognize ser. *Rupestria* as another genus is also supported by molecular phylogenetic data (Nikulin et al. 2016), but this needs a formal recognition. The data presented in this article might complement the research conducted so far on *S. rupestre*.

The formation of wall ingrowths and the presence of plasmodesmata with electron-dense dome were observed for the first time while studying the submicroscopical structure of *S. sediforme* antipodal cells. The presence of plasmodesmata in the outer walls of antipodals in *S. sediforme*, which is characteristic of Crassulaceae, led to the hypothesis that the occluded plasmodesmata may affect symplasmic communication during gametophyte development, as supposed for the seeds of *S. acre* L. (Wróbel-Marek et al. 2017). The function of antipodals is not yet defined or completely understood in the case of many angiosperms (Cass and Laurie 2001; Tekleyohans et al. 2017; Skinner and Sundaresan 2018). This actually supports the term ‘enigmatic features’ used by Tilton and Lersten (1981) in relation to the antipodal cells, one of the four cell types in a megagametophyte. The notation of new structural features can allow a better understanding of their role. The observations of ultrastructural, cytochemical and anatomical analyses of *S. sediforme* female gametophyte paved the way to the investigations performed on other species from ser. *Rupestria*. An additional argument favouring the choice of the species belonging to ser. *Rupestria* for further embryological studies was provided by the interesting report of Mauritzon (1933), who described several times the lack of cell wall formation between the antipodal nuclei in *S. anopetalum* DC. The above data and the unique structural features of *S. sediforme* antipodals indicate that the species from the genus ser. *Rupestria* are the appropriate objects for studies on antipodal cells. The ultrastructural features observed for the female gametophyte cells of *S*. *rupestre* are discussed in the light of the embryological data available for other angiosperm species and in relation to their expected functions.

The main aim of the present work is to describe the processes of megasporogenesis and megagametogenesis and the structure of the female gametophytic cells in *S. rupestre*. The findings of ultrastructural studies are supported by the results of cytochemical analyses, which enables a precise description of the substance characteristics and changes in their content. To the best of our knowledge, this is the first ultrastructural and cytochemical investigation on ovules conducted during the formation of female gametophyte in *S. rupestre*. Particular attention has been paid to the formation, differentiation and structure of the antipodals. The additional aim of the study is to comparatively analyze the embryological data collected during the previous and present research on the ovules of *Sedum* species during the development of female gametophyte. The hypothesis verified is that the species from ser. *Rupestria* (*S*. *rupestre* and *S*. *sediforme*) show common features during female gametophyte development (at the ultrastructural level and in the manner of development), similar to the finding noted during embryogenesis, which is related to the systematic position of these species.

## Materials and methods

### Plant material

For this study, ovules were isolated at various developmental stages from flower buds and flowers of *S. rupestre* L., a representative of the genus *Sedum*, belonging to the family Crassulaceae. The plant material collection was carried out during two growing seasons—2017/2018—in the natural habitats of Gdańsk (northern Poland) to individually observe the stages of megasporogenesis and megagametogenesis.

### Ovule clearing technique

A differential interference contrast (DIC) optic was used for the observation of cleared ovules. Samples were prepared according to the procedure described by Rojek et al. (2018). The isolated ovules were fixed in acetic alcohol. Subsequently, the samples were dehydrated in methanol followed by acidified 2,2-dimethoxypropane (DMP), and then preincubated in DMP:propylene oxide solutions (3:1 and 1:3, v/v) and finally in propylene oxide. The material was incubated in a mixture of cedar oil and propylene oxide (1:10, v/v). After the evaporation of propylene oxide, the cleared ovules were observed in a drop of pure cedar oil.

### Transmission electron microscopy and light microscopy observations

The ovules were prepared for light and transmission electron microscopy using the procedure described by Kozieradzka-Kiszkurno and Płachno (2013) for the analysis of Crassulaceae ovules. Briefly, the ovules were fixed in a mixture of 2.5% glutaraldehyde and 2.5% formaldehyde (prepared from paraformaldehyde) in 0.05 M cacodylate buffer (pH 7.0), at room temperature for 4 h. Next, they were rinsed in cacodylate buffer and post-fixed in 1% osmium tetroxide in cacodylate buffer overnight at 4 °C. The ovules were treated with 1% uranyl acetate (for 1 h), dehydrated in acetone series and embedded in Spurr’s epoxy resin (Spurr 1969). Finally, for transmission electron microscopic analysis, ultrathin, longitudinal sections were cut with a diamond knife on a Leica EM UC7 ultramicrotome and then post-stained with a saturated solution of uranyl acetate in 50% ethanol and 0.04% lead citrate. The sections were observed with an FEI Tecnai G^2^ Spirit TWIN/BioTWIN transmission electron microscope at 120 kV.

For light microscopic analysis, semi-thin, longitudinal sections were cut using a glass knife on a Sorvall MT 2B ultramicrotome. Cytochemical studies were carried out for control (with toluidine blue O) and test sections for the detection of proteins (with aniline blue black (ABB); Jensen 1962), lipids (with Sudan black B (SBB); Bronner 1975) and water-insoluble polysaccharides (with periodic acid-Schiff (PAS) reagent; Jensen 1962).

## Results

### Megasporogenesis and female gametophyte development

**Fig. 1:**
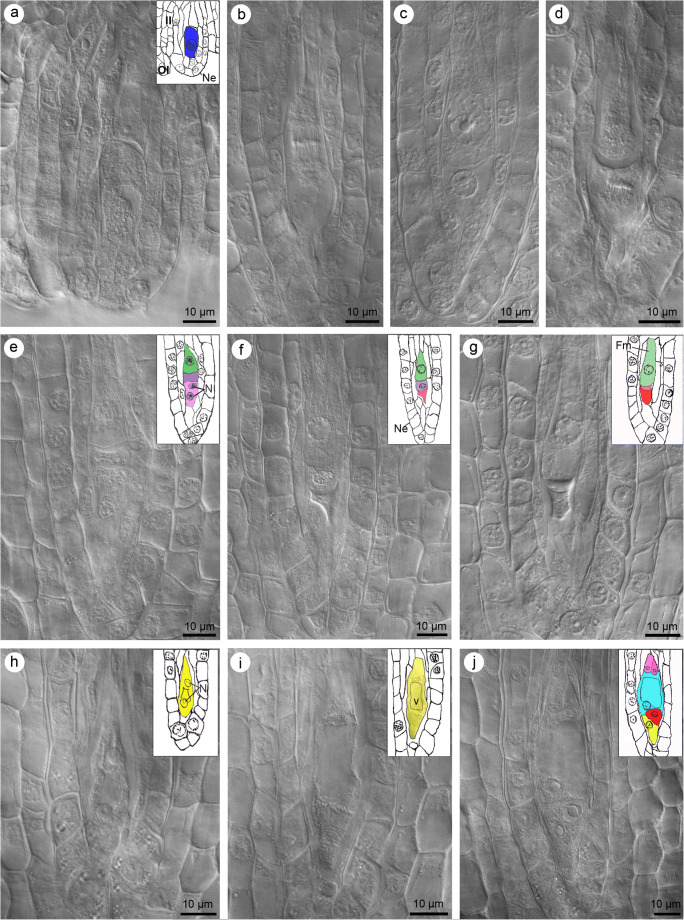
Megasporogenesis and megagametogenesis observed in photographs of cleared, unstained ovules of *Sedum rupestre* L. obtained using Nomarski differential interference contrast optic. Additional line drawings with the labels corresponding to the micrographs. **a** Anatropous and bitegmic ovule with megaspore mother cell. Nucellus with visible nucellar epidermis is covered by developing inner and outer integument. Drawing: megaspore mother cell (blue), nucellar epidermis (Ne), inner integument (II), outer integument (OI). **b** Megaspore mother cell during the first meiotic division – metaphase I. **c** Dyad stage. **d** Two cells of the dyad during the second meiotic division. **e** Linear triad of cells with one binucleate cell placed at the micropylar pole and two uninucleate megaspores located chalazally. Drawing: binucleate cell (pink), nucleus (N), megaspore placed in the middle of the triad (violet), chalazal megaspore (green). **f** Triad with a degenerating micropylar cell. Drawing: nucellar epidermis (Ne), degenerating cell (red), uninucleate megaspore placed in the middle of the triad (violet), chalazal megaspore (green). **g** Functional megaspore placed chalazally next to two degenerating cells located at the micropylar pole. Drawing: degenerating cell (red), chalazally placed functional megaspore (Fm, green). **h** Two-nucleate female gametophyte. Drawing: female gametophyte (yellow), nucleus (N). **i** Mitotic divisions visible at micropylar and chalazal ends of the developing female gametophyte. Drawing: female gametophyte (yellow), vacuole (V). **j** Seven-celled female gametophyte consisting of four cell types. Drawing: synergids (yellow), an egg cell (red), a central cell (blue), antipodal cells (pink)

First, the oval-shaped megaspore mother cell (MMC) initiated the process of megasporogenesis (Fig. 1a). The megasporocyte was found in the nucellus of the ovule, near the nucellar epidermis. MMC never came in direct contact with the epidermis (Fig. 1a), as one or two layers of cells always appeared and separated them from each other. During maturation, the ovule and MMC became elongated (Fig. 1b), followed by which the first and second meiotic divisions took place (Fig. 1b, d). As a result of the first meiotic division and cytokinesis (Fig. 1c), a dyad of cells was formed. The second meiotic division occurred in both micropylar and chalazal cells of the dyad (Fig. 1d). The time of division was not synchronized in both cells, and so one could observe the dyads with both cells during the nuclear divisions (Fig. 1d) and the triads with only micropylar cells during karyokinesis (not shown). Chalazal cells divided earlier than micropylar cells (Fig. 1d). Only triad formation was observed during megasporogenesis (Fig. 1e). The most micropylar cell of the triad was binucleate (as the only one), but it always degenerated (Fig. 1e). The degeneration process did not occur simultaneously in two non-functional cells. It began first in the most micropylar cell, followed by the cell in the middle (Fig. 1f). The linear triad formation was additionally confirmed by observations of two degenerating cells next to one FM (Fig. 1g). FM was mononucleate and the most chalazally placed cell in the triad (Fig. 1g).

During megagametogenesis, the first mitotic division of the nucleus within FM led to the formation of a two-nucleate female gametophyte (Fig. 1h). After the first mitosis, the vacuole separated the two newly formed nuclei which moved to the opposite poles of the gametophyte (Fig. 1h). Next, the nuclei at each pole divided mitotically twice (Fig. 1i), finally resulting in the formation of an eight-nucleate gametophyte. The vacuole was located in the central part of the coenocytic gametophyte. After cellularization of the coenocytic gametophyte, two synergids, one egg cell, one binucleate central cell and three mononucleate antipodals were formed (Fig. 1j).

### Anatomy, cytochemistry and ultrastructure of the female gametophyte

**Fig. 2:**
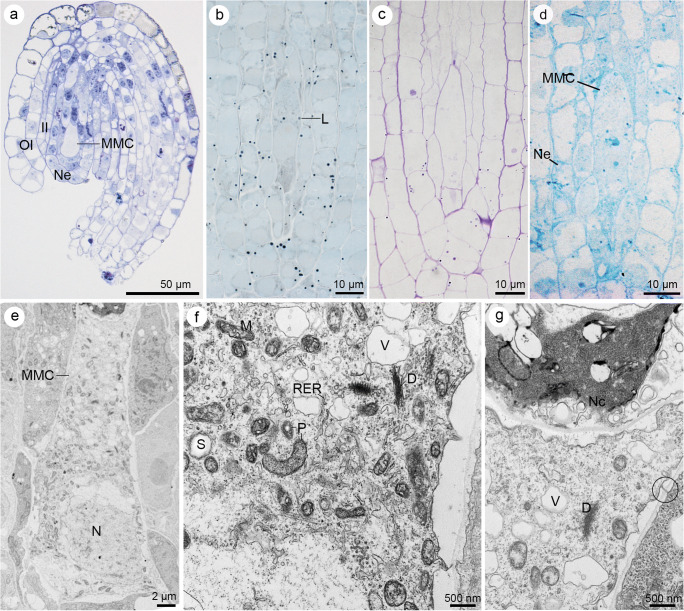
A megaspore mother cell stage in *Sedum rupestre* L. **a**–**d** Light microscopy observations—cytochemical staining results; **e**–**g** electron micrographs. **a** Megaspore mother cell (MMC) observed in the micropylar region of nucellus, but not in direct contact with nucellar epidermis (Ne). Oval-shaped MMC clearly differs from the surrounding nucellar cells in terms of size and shape. The outer (OI) and inner integument (II) cover the nucellus to different extent. **b** Distribution of lipid droplets (L) within the megaspore mother cell and the surrounding ovular cells visible after Sudan black B staining. **c** Section stained with periodic acid-Schiff reagent revealing the presence of insoluble polysaccharides. **d** Positive staining of aniline blue black revealing the presence of proteins. Megaspore mother cell (MMC), nucellar epidermis (Ne). **e** Ultrastructure of the megaspore mother cell (MMC) with nucleus (N) located in the micropylar region of the cell. **f** Magnified view of the central part of megasporocyte with dictyosomes (D), plastids (P) containing starch grains (S), mitochondria (M), vacuoles (V) and rough endoplasmic reticulum (RER). **g** Chalazal region of the megasporocyte with visible active dictyosomes (D) and small vacuoles (V) within the cytoplasm. Plasmodesmata (circle) are seen in the cell wall, which separate the MMC from nucellar cells (Nc)

**Fig. 3:**
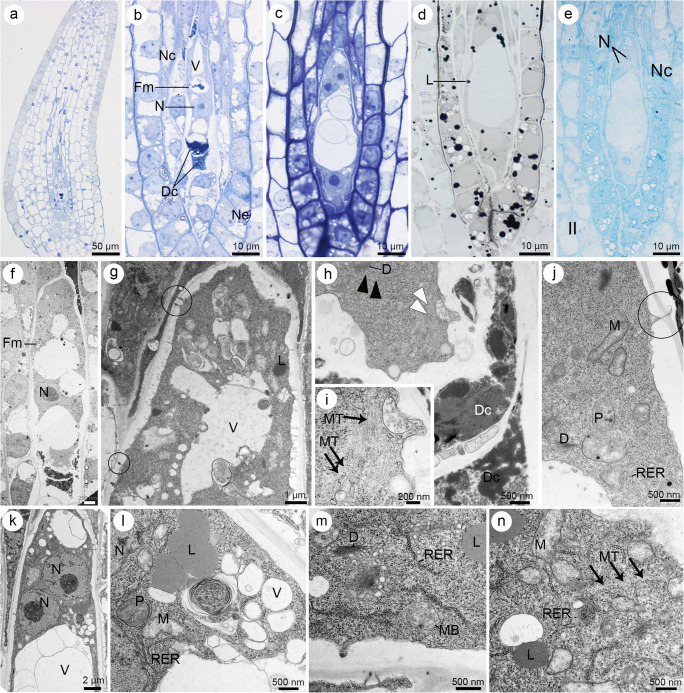
Formation of a functional megaspore and a coenocytic female gametophyte in *Sedum rupestre* L. **a**–**e** Light microscopic observations—cytochemical staining results; **f**–**n** electron micrographs. **a** Longitudinal section through the ovule during the differentiation of functional megaspore. **b** Magnified view of the micropylar part of the ovule from (**a**), showing visible functional megaspore (Fm)/one-nucleate (N) female gametophyte. Vacuoles (V) occur within the cytoplasm of the chalazally placed functional megaspore. Two degenerating non-functional cells (Dc) are located near the micropyle. Cells formed after meiosis are separated by several nucellar cells (Nc) from the nucellar epidermis (Ne). **c** Four-nucleate stage of embryo sac with visible degenerated nucellar cells located between the developing gametophyte and nucellar epidermis. **d** Longitudinal section through the coenocytic gametophyte stained with Sudan black B. Lipid droplets (L) are seen within the cytoplasm of the four-nucleate female gametophyte and the surrounding ovular cells. **e** Positive aniline blue black reaction observed in the cytoplasm of the four-nucleate (N) female gametophyte and micropylar nucellar cells (Nc), including nucellar epidermis; inner integument (II). **f** Functional megaspore (Fm)/one-nucleate female gametophyte placed in the vicinity of two degenerating, non-functional cells of the triad; nucleus (N). **g** Magnified view of the functional megaspore in the chalazal end, noted with vacuoles (V) and lipids (L) in the cytoplasm and plasmodesmata (circle) in the cell wall. **h** Magnified view of the micropylar region of the triad with active dictyosomes (D) and vesicles associated with them (black arrowhead), and microtubules (white arrowhead) located near the cell wall of the functional megaspore. Degenerating cells (Dc) are placed closer to the micropyle than the functional megaspore. **i** Magnified view of the cytoplasm of the functional megaspore from (h) demonstrating the presence of microtubules (MT, black arrows). **j** Mitochondria (M), plastids (P), dictyosomes (D) and profiles of rough endoplasmic reticulum (RER) visible in the cytoplasm of the functional megaspore, near the cell walls perforated by simple plasmodesmata (circle). **k** Chalazal pole of the four-nucleate megagametophyte with pronounced vacuole (V) and nuclei (N) in the cytoplasm. **l** Magnified view of the cytoplasm of the four-nucleate megagametophyte from (k), noted with vacuoles (V), nucleus (N), plastids (P), mitochondria (M) lipid droplets (L) and profiles of rough endoplasmic reticulum (RER). **m** Magnified view of the cytoplasm of the four-nucleate megagametophyte from (k), with visibly present active dictyosomes (D), microbodies (MB), profiles of rough endoplasmic reticulum (RER) and lipid droplets (L). Some electron-dense dome and RER are seen near the cell wall where plasmodesmata occur. **n** Microtubules (MT, black arrows), mitochondria (M), profiles of rough endoplasmic reticulum (RER) and lipid droplets (L) observed in the cytoplasm of the four-nucleate megagametophyte from (**k**)

The ovule of *S. rupestre* was anatropous and crassinucellate. Two integuments covered the nucellus (bitegmic ovule). However, at the early stage of MMC formation, when its shape appeared more oval than elongated, the inner and outer integuments partly covered the nucellus. During ovule development, the outer integument grew faster compared to the inner one, as a result of which they had a different extent (Fig. 2a). Finally, the integuments covered the entire nucellus forming the micropyle at the top. Cells arranged in two layers developed both inner and outer integuments (Fig. 3a).

Cytochemical staining showed that the MMC contains lipids (Fig. 2b), insoluble polysaccharides (Fig. 2c) and proteins (Fig. 2d). Mitochondria, nucleus, the profiles of endoplasmic reticulum (ER), small vacuoles and vesicle-producing dictyosomes could be distinguished in the cytoplasm of megasporocyte (Fig. 2e–g). Small lipid droplets were seen scattered in the cytoplasm of MMC, next to the plastids filled with starch granules (Fig. 2f). Simple plasmodesmata occurred in the cell walls of MMC, but mainly at the chalazal pole (Fig. 2g).

Semi-thin (Fig. 3a, b) and ultra-thin (Fig. 3f) sections showed that a triad of cells arranged in one line along the micropylar–chalazal axis. The formation of cell wall was not observed within the micropylar dyad cell after karyokinesis. Several vacuoles occurred within the developing FM/one-nucleate female gametophyte (Fig. 3b, f), which started to coalesce with each other (Fig. 3f, g) forming a bigger one, centrally located in the two-nucleate and four-nucleate megagametophyte (Fig. 3c–e). Moreover, in the chalazal region of the four-nucleate female gametophyte, an additional smaller vacuole could be observed (Fig. 3c). In coenocytic female gametophyte, the central vacuole separated and moved to the nuclei in two opposite poles (chalazal and micropylar) which were formed after three series of mitosis, not followed by the formation of cell wall (Fig. 3c–e).

**Fig. 4:**
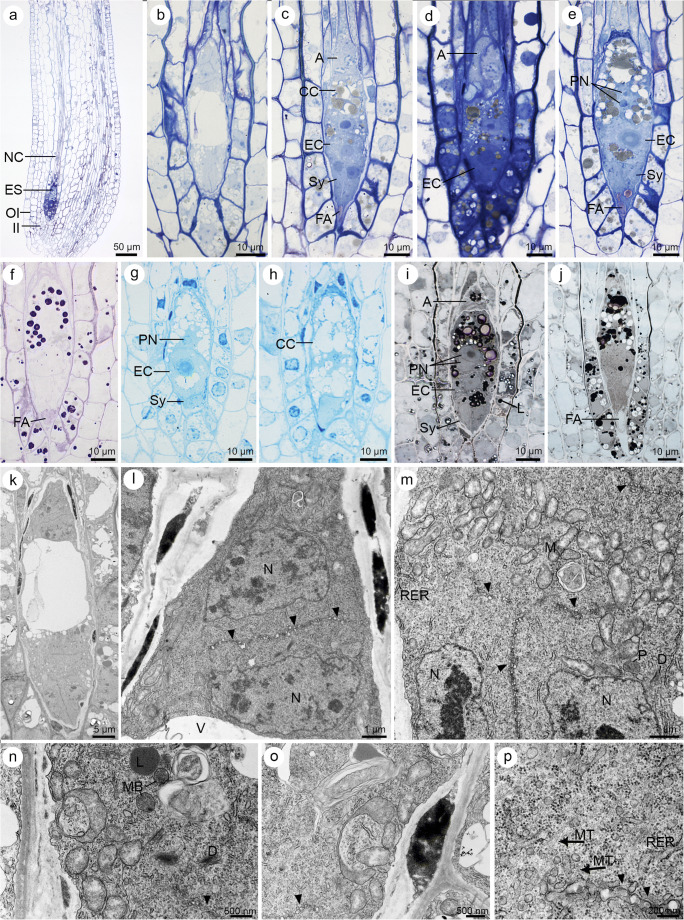
Cellularization of the coenocytic female gametophyte and formation of seven-celled embryo sac in *Sedum rupestre* L. **a**–**j** Light microscopic observations—cytochemical staining results; **k**–**p** electron micrographs. **a** Longitudinal section through the ovule and embryo sac (ES). Outer (OI) and inner integument (II) cover the nucellar cells (NC). **b** Female gametophyte during the formation of cell walls. **c** Seven-celled female gametophyte with visible three antipodal cells (A), one central cell (CC), one egg cell (EC) and two synergids (Sy) having formed filiform apparatus (FA). **d** Obvious antipodal cells (A) observed at the chalazal pole of the megagametophyte. An egg cell (EC) is located at the opposite, micropylar pole. **e** Synergids (Sy) with filiform apparatus (FA), one egg cell (EC) and a central cell having two polar nuclei (PN) visible in the vicinity of the degenerating antipodal cells. **f** Detection of polysaccharides in a filiform apparatus (FA) and megagametophytic cells. **g** Positive staining with aniline blue black (ABB) observed in both synergids (Sy), an egg cell (EC) and the cytoplasm of central cell showing two polar nuclei (PN). **h** Presence of proteins recorded within the antipodal cells after ABB staining; central cell (CC). **i** Presence of lipid droplets (L) in seven-celled megagametophyte visualized by Sudan black B (SBB) staining. A semi-thin section is showing all types of female gametophytic cells: antipodals (A), a central cell with two polar nuclei (PN), an egg cell (EC) and synergids (Sy) with accumulated lipids. **j** Lipid droplets seen in the nucellar epidermis and female gametophytic cells. A filiform apparatus (FA) is not stained with SBB. **k** Ultrastructure of the female gametophyte during the formation of cell walls. **l** Magnified view of the chalazal part of the female gametophyte during the formation of cell wall (black arrowhead) with visible nuclei (N) and vacuole (V). **m** Magnified view of the micropylar part of the female gametophyte during the formation of cell wall (black arrowhead). Nuclei (N), mitochondria (M), plastids (P), dictyosomes (D) and profiles of rough endoplasmic reticulum (RER) are present in the cytoplasm. **n** Ultrastructure of the cytoplasm during megagametophyte cellularization, showing visible microbodies (MB), lipid droplets (L), dictyosomes (D) and fragments of cell wall (black arrowhead). **o** Ultrastructure of the megagametophyte cytoplasm during the formation of cell walls (black arrowhead). **p** Magnified view of the cytoplasm near the formed cell wall (black arrowhead) showing the presence of microtubules (MT, black arrow), vesicles and profiles of rough endoplasmic reticulum (RER)

Staining revealed the presence of proteins and lipids in coenocytic megagametophyte (Fig. 3d, e). Lipid droplets occurred within FM (Fig. 3g), but their accumulation increased during megagametogenesis (Fig. 3k–n) particularly within the cellular gametophyte (Fig. 4i, j). Ultrastructural analysis showed that similar to lipid droplets, few starch granules also occurred within FM (not shown). Moreover, the storage materials were detected in the surrounding ovular cells, especially in the micropylar region of the nucellus epidermal cells. The presence of starch grains and lipid granules and a positive ABB staining were observed in the micropylar region of nucellar epidermis from the beginning of the megasporogenesis (Fig. 2b–d) until the formation of the embryo sac (Fig. 3d, e; 4f–j)—the content of the lipid droplets, starch granules and proteins increased. Simple plasmodesmata were noted in the FM walls, but in the chalazal region (Fig. 3g, j). The microtubules occurred near the cell walls and the active dictyosomes which produced vesicles within the cytoplasm of FM (Fig. 3h, i) and the four-nucleate female gametophyte (Fig. 3n). Nuclei, plastids, profiles of rough ER (RER), mitochondria and microbodies were also detected in the cytoplasm of the coenocytic female gametophyte (Fig. 3j–n). ER was also found near the plasmodesmata and electron-dense material (Fig. 3m), adjacent to the cell wall in the regions where plasmodesmata occurred.

The formation of the cellular gametophyte (Fig. 4a) was preceded by that of cell wall (Fig. 4b). Cellularization resulted in the presence of three one-nucleate antipodal cells in the chalazal region of megagametophyte, one central cell in the central part of the embryo sac, one egg cell and two synergids arranged nearest to the micropyle (Fig. 4c–e). The filiform apparatus was found in the micropylar region of both synergids (Fig. 4e) and was stained by PAS reagent (Fig. 4f) and ABB (not shown), but not by SBB (Fig. 4j). The central cell was the only one binucleate cell within the megagametophyte. Two polar nuclei were observed before (Fig. 4c, i) and after the degeneration process began within the antipodal cells (Fig. 4e). The highest content of starch granules was detected first within the central cell and only later in the egg cell, both synergids (Fig. 4f) and the antipodals. Positive protein staining was observed in all gametophytic cells (Fig. 4g, h). The lipid droplets were also detected in all the gametophytic cells, but only the central cell showed the greatest accumulation (Fig. 4i, j). Cuticle was not observed on the micropylar surfaces of the nucellar epidermis and the female gametophyte (Fig. 4i, j).

Ultrastructural analysis of the embryo sac during the formation of cell wall showed that the process took place in the chalazal and micropylar regions simultaneously at both poles of the same gametophyte (Fig. 4k). However, this observation did not explain whether the cell wall formation process was synchronized. The newly formed cell walls separated the nuclei from each other (Fig. 4l, m). In the chalazal region of the gametophyte, the electron-dense material was locally concentrated near the outer cell wall (Fig. 4l). At this stage of development, the cytoplasm had spherical- to ellipsoidal-shaped mitochondria, some active dictyosomes, short profiles of RER, microbodies and plastids (Fig. 4l–o). Microtubules and numerous dictyosomes with vesicles were observed in the vicinity of the forming cell wall (Fig. 4n, p).

**Fig. 5:**
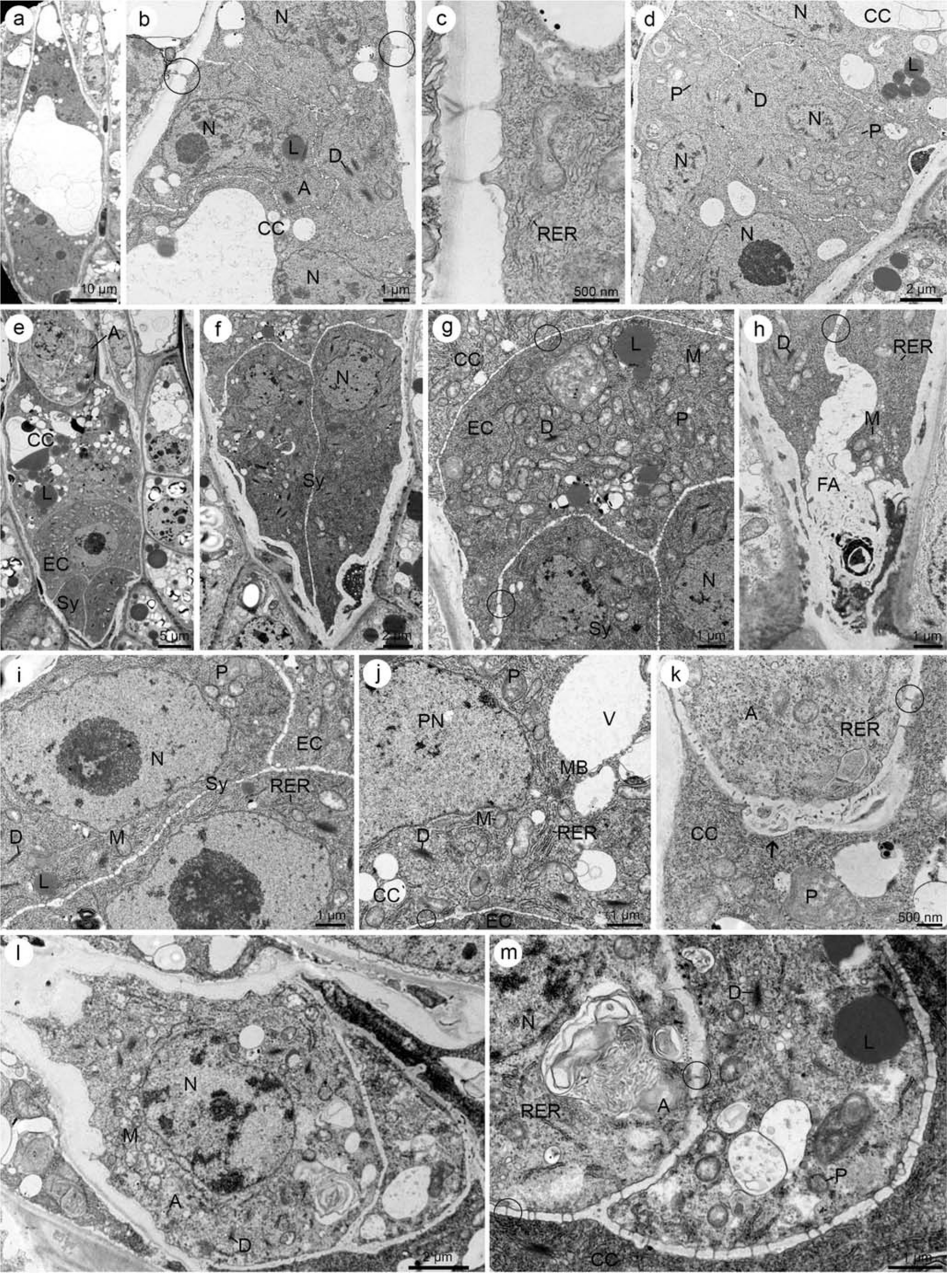
Electron micrographs showing the ultrastructure of the seven-celled female gametophyte in *Sedum rupestre* L. **a**–**d** After cellularization and **e**–**m** during cell differentiation. **a** Longitudinal section through the seven-celled megagametophyte. **b** Three mononucleate antipodal cells (A) with non-differentiated cytoplasm located in the chalazal region of the female gametophyte next to the central cell (CC) after cellularization. Nuclei (N), lipid droplets (L), dictyosomes (D) and plasmodesmata with electron-dense dome (circle) are observed. **c** Magnified view of the outer wall of the antipodal cell from (**b**) showing visible simple and branched plasmodesmata with adjacent electron-dense dome and profiles of rough endoplasmic reticulum (RER). **d** Magnified view of the micropylar region of the seven-celled female gametophyte. Nuclei (N), plastids (P), dictyosomes (D) and lipid droplets (L) are observed in the cytoplasm of the egg apparatus and central cell (CC). The cytoplasm is not fully differentiated. **e** Longitudinal section through the seven-celled megagametophyte during the cell differentiation. Synergids (Sy), an egg cell (EC), antipodal cells (A) and central cell (CC) with numerous lipid droplets (L) are observed. **f** Ultrastructure of synergids (Sy) cytoplasm with visible nuclei (N) located in the chalazal regions of the cells. **g** Presence of plasmodesmata (circle) noted in the walls separating an egg cell (EC) and a central cell (CC) as well as an egg cell and synergids (Sy). Nuclei (N), mitochondria (M), plastids (P), dictyosomes (D) and lipid droplets (L) are observed. **h** Mitochondria (M), dictyosomes (D) and profiles of rough endoplasmic reticulum (RER) observed in the vicinity of a filiform apparatus (FA). Plasmodesmata (circle) are present in walls separating both synergids. **i** Magnified view of the chalazal region of synergids (Sy) and the fragment of an egg cell (EC) showing the presence of nuclei (N), mitochondria (M), plastids (P), dictyosomes (D), profiles of rough endoplasmic reticulum (RER) and lipid droplets (L) in both synergids. **j** Cytoplasm rich in mitochondria (M), plastids (P), dictyosomes (D), profiles of rough endoplasmic reticulum (RER), some microbodies (MB) and vacuoles (V) near the polar nuclei (PN). Plasmodesmata (circle) are noted in the walls separating an egg cell (EC) and a central cell (CC). **k** A cell wall separating the central cell (CC) and antipodal cells (A) rich in simple plasmodesmata (circle). Plastids (P), profiles of rough endoplasmic reticulum (RER) and fragments of the second antipodal cell are visible (black arrow). **l** A cell wall of antipodals (A) not having exactly the same thickness over the entire length; nucleus (N), mitochondria (M), dictyosomes (D). **m** Numerous plasmodesmata (circle) occurring in the wall between antipodals (A) and the wall between an antipodal and a central cell (CC). Nucleus (N), dictyosomes (D), plastids (P), profiles of rough endoplasmic reticulum (RER) and lipid drop (L) are observed within the cytoplasm of antipodals

Individual cells formed at the end of the cellularization process did not differ from each other in relation to the electron density in their cytoplasm (Fig. 5a, b, d). However, antipodal cells had plasmodesmata with electron-dense material in the chalazal walls (Fig. 5b, c), while the central cell had a large central vacuole. In addition, the position and number of nuclei within the cells varied (Fig. 5a, b, d). Gametophytic cells were mononuclear with the exception of the central cell. The two polar nuclei of the central cell were located initially at the opposite poles, but during development, they moved towards the egg cell. The ultrastructure of the gametophytic cells changed as they matured. The cells became rich in organelles. In the egg cell, the nucleus was located in the chalazal part, while in each synergid cell, it was placed centrally or chalazally (Fig. 5e–g). Plasmodesmata were visible in the walls separating the egg cell and central cell, as well as in those separating the egg cell and synergids (Fig. 5g). Plastids, lipid droplets, dictyosomes, mitochondria and some profiles of RER were observed in the cytoplasm of the egg cell and both synergids (Fig. 5g–i). However, the filiform apparatus was observed only in the micropylar part of synergids, in the vicinity of which mitochondria and small vesicles (appeared to have originated from dictyosomes) were found (Fig. 5h). Organelles such as mitochondria and profiles of RER were distributed throughout the cytoplasm of synergids; however, they were located parallel to the long axis in their micropylar part. Plasmodesmata were also found in the walls that separated both synergids (Fig. 5h). The central cell contained two polar nuclei and scattered plastids, mitochondria, microbodies, profiles of RER, active dictyosomes and few small vacuoles throughout the cytoplasm (Fig. 5j, k). Simultaneously, some RER cisternae were found stacked in three or four layers next to the plastids, while mitochondria were found near the polar nuclei. The wall between the central cell and antipodals was rich in simple plasmodesmata. However, the most chalazal cell wall of the gametophyte, which separated the antipodals from the adjacent nucellus, appeared thicker compared to the other antipodal cell walls (Fig. 5k–m).

**Fig. 6:**
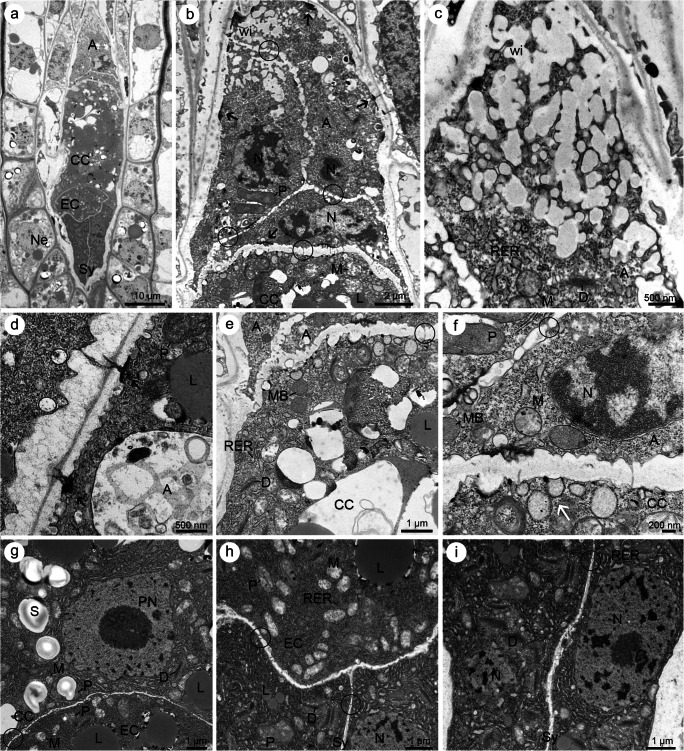
Electron micrographs showing the ultrastructure of the seven-celled female gametophyte in *Sedum rupestre* L. **a** Longitudinal section through the seven-celled megagametophyte. Three antipodal cells (A), one central cell (CC), an egg cell (EC) and two synergids (Sy) are visible. Nucellar epidermis (Ne) is adhering locally to the female gametophyte. **b** Chalazal pole of the female gametophyte having three visible differentiated antipodal cells (A). Simple plasmodesmata (circle) are observed in the cell walls separating the antipodal cells from each other and the antipodals from a central cell (CC). An electron-dense material (arrow) is observed near some cell walls and near plasmodesmata located in the outer cell walls of antipodals, separating these cells from nucellar cells. Wall ingrowths (wi), nuclei (N), plastids (P), mitochondria (M) and lipid droplets (L) are noted. **c** Presence of wall ingrowths (wi) observed in the outer walls of antipodal cells (A). Profiles of rough endoplasmic reticulum (RER), mitochondria (M) and dictyosomes (D) occur in the vicinity of the wall ingrowths. **d** Simple and branched plasmodesmata with electron-dense dome (arrow) located in the outer walls of antipodal cells (A) observed under high magnification; lipid drop (L), plastid (P). **e** A part of the cytoplasm of the central cell (CC) and antipodal cells (A) noted with plasmodesmata (circle), microbodies (MB), lipid droplets (L), dictyosomes (D) and profiles of rough endoplasmic reticulum (RER). **f** Magnified view of the fragment from (e) showing plasmodesmata (circle), plastids (P), mitochondria (M), microbodies (MB) and nucleus (N) in antipodal cells (A). Some vesicles (arrow) are seen in the cytoplasm of the central cell (CC). **g** Cytoplasm of the central cell (CC), located near-polar nuclei (PN), having plastids (P) with accumulated starch grains (S), lipid droplets (L), mitochondria (M) and dictyosomes (D). Simple plasmodesmata (circle) are observed in the walls separating the central cell and an egg cell (EC). **h** Chalazal part of the synergids (Sy) and a fragment of the cytoplasm of an egg cell (EC). Nuclei (N), numerous active dictyosomes (D), some lipid droplets (L) and plastids (P) are seen near the profiles of rough endoplasmic reticulum (RER) in the cytoplasm of synergids. Plastids, mitochondria (M), lipid droplets and some profiles of rough endoplasmic reticulum are also noted in the cytoplasm of the egg cell; plasmodesmata (circle). **i** Magnified view of the synergids (Sy) with visibly active cytoplasm. Nuclei (N), dictyosomes (D), profiles of rough endoplasmic reticulum (RER) and plasmodesmata (circle) are observed

Plastids, mostly singular profiles of RER, mitochondria, dictyosomes and lipid droplets occurred in the cytoplasm of the antipodal cells (Fig. 5l, m; 6a–c). During the development of female gametophyte, a prominent formation of wall ingrowths was observed in the chalazal region of these cells (Fig. 6a–c). Cytoplasm with mitochondria, profiles of RER and active dictyosomes were observed in close contact with the fine transfer-wall ingrowths which allowed them to penetrate the surrounding areas (Fig. 6c). Simple plasmodesmata were noted between all the female gametophytic cells including antipodals (Fig. 6b). However, the plasmodesmata in the outer walls of the antipodals, bordered by sporophytic cells, showed a different structure, as they perforated the walls and appeared additionally branched (Fig. 6b, d). Moreover, the electron-dense material adjacent to them formed the cytoplasm side of the antipodals (Fig. 6b, d). Sometimes, the co-localization of the electron-dense material and walls separated the antipodals from each other (Fig. 6b, e, f).

**Fig. 7:**
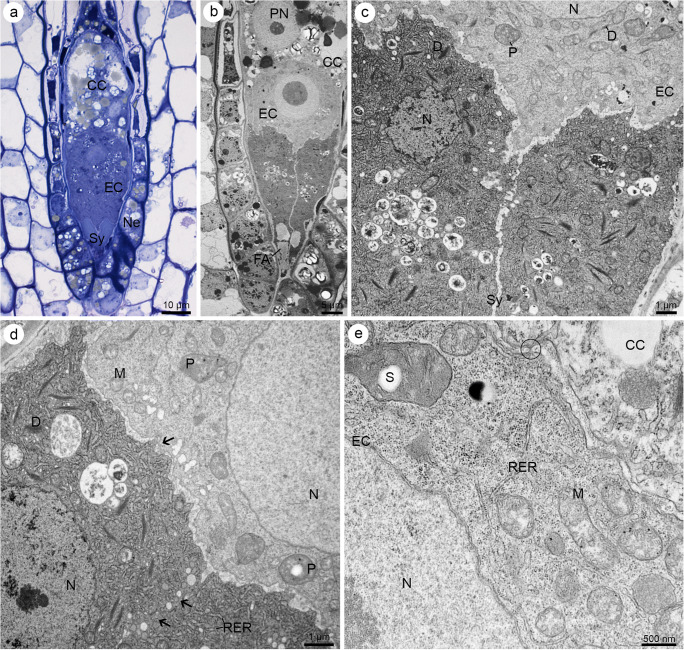
Mature female gametophyte of *Sedum rupestre* L. **a** Light micrograph; **b**–**e** electron micrographs. **a** Longitudinal section through the mature female gametophyte showing a central cell (CC), an egg cell (EC) and two synergids (Sy); nucellar epidermis (Ne). **b** Ultrastructure of the female gametophyte from (**a**) with visible filiform apparatus (FA) in the micropylar part of synergids, polar nucleus (PN) within a central cell (CC) and an egg cell (EC). Synergids have visibly more electron-dense cytoplasm than the other megagametophytic cells. **c** Magnified view of the chalazal part of the synergids (Sy), near an egg cell (EC), with visible nucleus (N) and active dictyosomes (D) within the cytoplasm; plastid (P). **d**. Chalazal pole of the synergids with locally thinner or absent cell wall (black arrow). Electron-dense cytoplasm of the synergids contains active dictyosomes (D) and abundant profiles of rough endoplasmic reticulum (RER); nucleus (N), plastid (P), mitochondria (M). **e** Cytoplasm of a central cell (CC) and an egg cell (EC) observed under high magnification. Plasmodesmata (circle) occur in the cell wall separating the central cell and an egg cell. Plastids with starch grains (S), mitochondria (M), nucleus (N) and profiles of rough endoplasmic reticulum (RER) are noted in the cytoplasm of the egg cell

During the development of the female gametophyte, when the antipodals were present, the cytoplasm of the central cell contained microbodies, profiles of RER, active dictyosomes, lipid droplets, starch grains accumulated within some plastids and mitochondria next to the two polar nuclei (Fig. 6e, g). The ultrastructure of the synergids showed that they were more active compared to the earlier stages of development and the other female gametophytic cells (Fig. 6h, i). Two ultrastructurally similar synergids were observed in the micropylar region of the embryo sac. Their cytoplasm changed in relation to the nature of the organelles, including the predominantly increased content of RER profiles, increased dictyosomes activity and electron density (Fig. 6h, i). This phenomenon was also clearly visible at a later stage of female gametophyte development, after the antipodals started to degenerate (Fig. 7a–d). Then, the synergids became clearly distinct from other gametophytic cells. Additionally, vacuoles of various sizes gathered close together within their cytoplasm, containing fine fibrillar material (Fig. 7b–d). A relatively higher density of the cytoplasm and content of organelles indicated the greater metabolic activity of the synergids than the egg cell (Fig. 7b–d). The cytoplasm density of the central cell also differed from that of the synergids. However, the central cell could be clearly distinguished by the presence of storage materials such as huge lipid droplets and numerous starch grains (Fig. 7b). The filiform apparatus was still noted in the micropylar region of synergids (Fig. 7a, b), while the chalazal walls of these cells became thinner and even invisible in some places (Fig. 7c, d). Plasmodesmata occurred in the wall that separated the egg cell from the central cell (Fig. 7e). The female gametophyte elongated during development*.*

## Discussion

The ovule of *S*. *rupestre* was anatropous—similar to most angiosperms and Crassulaceae species (Rombach 1911; Mauritzon 1933; Thiede and Eggli 2007; Bhojwani et al. 2015), crassinucellate—similar to other representatives of Sempervivoideae, next to Kalachoideae (Thiede and Eggli 2007), and bitegmic. In Crassulaceae, both the integuments have been found to be two-layered (Rombach 1911; Thiede and Eggli 2007), with their growth rate varying depending on the species (Mauritzon 1933). In *S. rupestre*, the outer integument was found to grow faster than the inner one, as observed in the case of *S. sediforme* (Brzezicka and Kozieradzka-Kiszkurno 2019), *S. hispanicum* (Brzezicka and Kozieradzka-Kiszkurno 2018) and most Crassulaceae species (Rombach 1911; Mauritzon 1933). Moreover, two integuments formed the micropyle in the studied species, as has been commonly described for the representatives of the Crassulaceae family. Contrasting observations have been reported in the case of, for example, *Sedum kirilowii*, *Echeveria pulverulenta* (the outer integument grows slower compared to the inner one) or *Echeveria gibbiflora* and *Rosularia elymaitica* (the inner integument stops growing and the micropyle is formed only by the outer one) (Mauritzon 1933). However, the elongation degree of ovule and megagametophyte in *S. rupestre* was similar to that seen in *S. sediforme*, which is a closely related species (Nikulin et al. 2016).

Furthermore, similar to most of the other representatives of the genus *Sedum* and family Crassulaceae, *S. rupestre* showed the monosporic type of megasporogenesis and *Polygonum*-type development of female gametophyte. In angiosperms, the most often observed event of megasporogenesis is the formation of a megaspore tetrad. In the studied species, a triad was noted, similar to three other *Sedum* representatives (*S. sediforme*, *S. caeruleum* Vahl, *S. annuum* L.) (Mauritzon 1933; Brzezicka and Kozieradzka-Kiszkurno 2019). However, in the last two species, the tetrad of megaspores is also formed as a result of megasporogenesis. In *S. sediforme* and *S. rupestre*, triad formation was the one event observed after megasporogenesis, since the wall formation was not recorded in the micropylar dyad cell. This situation (only the formation of a triad with a binucleate micropylar cell during megasporogenesis) has not been observed in other Crassulaceae species studied so far. If a triad with a binucleate micropylar cell formation occurs in other Crassulaceae, then it is an additional result of megasporogenesis: (1) next to the linear tetrad or (2) next to the triad made of uninucleate cells (only in *Rosularia sempervivum* Berger) or (3) next to both the uninucleate-cell triad and tetrad. In *Umbilicus intermedius* and *Echeveria rosei*, only the triad composed of mononucleate cells was observed after megasporogenesis. Interestingly, tetrad formation is the only megasporogenesis event described for *Sedum anopetalum* (Mauritzon 1933) species of ser. *Rupestria*; therefore, it cannot be currently determined whether the formation of triad with a binucleate micropylar cell is a common feature for all the representatives of ser. *Rupestria*. An in-depth analysis including verification of whether the *Polygonum*-type development of the female gametophyte is modified in the same way among the other species of ser. *Rupestria* seems interesting. Moreover, this analysis may be considered as necessary and reasonable especially because of the number of morphological, anatomical and ultrastructural similarities found among its representatives.

Some angiosperms, including *Sedum* and Crassulaceae species, show the presence of additional structures facilitating the absorption and transport of nutrition from surrounding tissues. In Crassulaceae, haustoria formation was observed during megasporogenesis and during the formation of the mature embryo sac in *Sedum sempervivoides* and *Rosularia pallida* where synergid haustoria were formed and in *Sedum fabaria* Schinz and Keller where antipodal haustoria were formed (Mauritzon 1933; Johri et al. 1992; Wojciechowicz and Samardakiewicz 1998). However, haustoria formation was not observed in the studied species during the development of female gametophyte, but wall ingrowths occurred in two opposite poles of the embryo sac—micropylar and chalazal. They were present within the synergids and antipodals. Cells with wall ingrowths and protuberances (transfer cells) are useful for short-distance transport (absorption, secretion or exchange) in angiosperms (Gunning and Pate 1969; Pate and Gunning 1972) including *S. rupestre.* In addition, the presence of wall ingrowths contributes to an increase in the surface of the plasma membrane, regardless of their structural type and morphology, but to a different extent (Pate and Gunning 1972; Johri and Ambegaokar 1984). In *S. rupestre*, the occurrence of wall ingrowths in the micropylar (synergids) and chalazal (antipodal cells) part of the female gametophyte showed that in these regions greater diffusive flux occurs, simultaneously indicating the places where most active transport of solute took place. Nutrients can be transported between sporophyte, ovular tissues and developing gametophyte via transfer cells (Pate and Gunning 1972; Johri and Ambegaokar 1984). In addition, the ovular tissues not only provide nutrients but also take part in their transfer (Willemse and van Went 1984). The nutritional function related with the transport of nutrients towards the megagametophyte through wall ingrowths has been described as quite possible in both the antipodal cells and the synergids (Willemse and van Went 1984; Johri and Ambegaokar 1984; Reiser and Fischer 1993; Yang 2006; Punwani and Drews 2008). This was also supported by the sites (micropylar and chalazal) of haustoria formation in Crassulaceae during the development of female gametophyte. The ultrastructure of the cytoplasm and the anatomical features such as wall ingrowths show that the antipodals of *S. rupestre* are synthetically active and participate in material transport, similar to the so-called transfer cells (Gunning and Pate 1969). The cytoplasm of antipodals became dense and rich in organelles such as ribosomes, microbodies, active multicisternal dictyosomes and vesicles derived from the profiles of ER, and extensive wall ingrowths formed at the chalazal part of the cells. In addition, accumulation of lipid droplets and the presence of small starch grains and proteins were noted. Antipodals also contained such materials in other angiosperms (Reiser and Fischer 1993), including *Ornithogalum caudatum* (Tilton and Lersten 1981) and *S. oleracea* (Wilms 1980). The structural features described for the antipodals of *S. rupestre* indicate the high metabolic activity of these cells. The results obtained during this study and the data collected so far indicate that *Sedum* antipodals can participate in the transfer of nutrients from nucellus.

The function of antipodal cells is currently unknown in many plants, including *Arabidopsis* which is the model plant (Tekleyohans et al. 2017; Skinner and Sundaresan 2018). A lack of general conclusions and clearly defined function is associated with much variation noted regarding the antipodals in angiosperms (Willemse and van Went 1984). In *S. rupestre*, the antipodals degenerated during the maturation of megagametophyte, as has been observed in *Passiflora caerulea* (García et al. 2003), *Downingia* (Kaplan 1969) and many other taxa of dicotyledons (Willemse and van Went 1984). In addition, the anatomical and ultrastructural structure of the antipodals changed in the studied species during the development of megagametophyte and cell differentiation. Initially, after the formation of cell wall in the coenocytic gametophyte, the cytoplasm of antipodal cells appeared similar to that of the other female gametophytic cells. A similar observation was reported for *S. oleracea*, in which the cells exhibited the same ultrastructure in young megagametophyte (Wilms 1980). A distinct feature of the antipodals in *S*. *rupestre* was the presence of plasmodesmata with electron-dense material on the cell side. It can be assumed that those plasmodesmata present in the outer walls of antipodals are functional. The results obtained from studies on *Arabidopsis* ovules also suggest the presence of symplasmic connections between antipodal cells and the neighbouring nucellar cells (Lawit et al. 2013). Moreover, it is suggested that chalazal nucellar cells provide an additional antipodal specification factor via symplasmic connections (Skinner and Sundaresan 2018). Almost identical ultrastructural data on the arrangement and structure of plasmodesmata were provided for *S*. *sediforme* (Brzezicka and Kozieradzka-Kiszkurno 2019). Similar findings were observed in *Spinacia oleracea* L. (Wilms 1980) and *Capsella* (Schulz and Jensen 1971), which exhibited the presence of simple plasmodesmata in the outer walls of antipodals. The presence of plasmodesmata facilitates the exchange of substances such as nutrients and developmental signals (Benitez-Alfonso 2014 and literature therein). The data of the present study also confirm that antipodal cells of *S*. *rupestre* exhibit both symplasmic and apoplasmic connections with the surrounding nucellar cells.

Correct differentiation and specification of the female gametophytic cell fates is necessary for the proper functioning of embryo sac cells and consequently for double fertilization (Skinner and Sundaresan 2018). The main function of the synergids is to secrete pollen tube attractant proteins through their filiform apparatus, which is likely involved in this process as well as in pollen tube reception (Punwani and Drews 2008; Higashiyama and Yang 2017; Tekleyohans et al. 2017). However, two female gametes are not neutral for the process of pollen tube guidance, since they control the development and function of the synergids, including the persistent one (Higashiyama and Yang 2017). The ultrastructure of the synergids and cell differentiation in *S. rupestre* showed that these cells become more active during development and perform secretion in the mature female gametophyte. Both synergid cells were ultrastructurally similar in the studied species, which is in line with the statement that these two participate in attractant secretion. Moreover, the presence of a correctly formed filiform apparatus in the synergids of *S. rupestre* indicated that these cells have the ability to attract pollen tube. This supports the hypothesis that the correct formation of the filiform apparatus correlates with the attractive function of the synergids (Tekleyohans et al. 2017).

In *S. rupestre*, both synergids were found to be highly active cells, and also contained secretory organelles in the vicinity of the filiform apparatus. The nucellar tissue in this species was separated from the integuments by a cuticle, which was conspicuous along the entire length of the nucellus but was visibly less pronounced or totally absent in its micropylar part. However, the cuticle was not observed in the micropylar region of the female gametophyte, similar to that reported for *Paphiopedilum delenatii* in which the absence of cuticle was also stated for the chalazal side (Lee and Yeung 2012). Lack of cuticle at the micropylar pole of megagametophyte can be related to synergids activity, which is facilitated by their filiform apparatus. It was stated that the micropylar region is not cutinized in most plants (Berger and Erdelska 1973). All the female gametophytic cells of the studied species were stained by ABB and PAS, which showed that their cytoplasm is rich in proteins and insoluble polysaccharides. Similar observations were made with respect to lipids, which were noted as droplets in all cells of the embryo sac in the species. The presence of lipid bodies and starch accumulation were also observed in the synergids of *A. hypochondriacus*, which probably play a role in nutrient absorption (Coimbra and Salema 1999). Participation of synergid cells in absorption also cannot be excluded in *S. rupestre*, since some lipid bodies and starch grains were noted in synergids cytoplasm in addition to lack of cuticle at the micropylar pole of embryo sac, where the formation of filiform apparatus occurred.

The habitats of most Crassulaceae are semi-arid/arid and rocky regions. The representatives of this family are disturbed worldwide, but these species, especially *Sedum*, are predominantly found in the areas of the northern hemisphere (temperate and subtropical zones) and Africa. However, only a few *Sedum* species occur in the central-east regions of Africa and in areas of South America (Eggli 2003; Hart and Bleij 2003; Thiede and Eggli 2007). *Sedum hispanicum* and the representatives of ser. *Rupestria* are Euro-Mediterranean species (Thiede and Eggli 2007; Gallo 2012), classified under other clades (Nikulin et al. 2016). A comparative analysis of the embryological features observed during ultrastructural and cytochemical studies of three *Sedum* species—*S. hispanicum*, *S. sediforme*, *S. rupestre*—revealed that *S. rupestre* has more similarity to *S. sediforme* than *S. hispanicum*. These embryological findings are consistent with morphological characteristics, molecular phylogenetic data and earlier observations made during embryogenesis (Thiede and Eggli 2007; Nikulin et al. 2016; Czaplejewicz and Kozieradzka-Kiszkurno 2013; Kozieradzka-Kiszkurno et al. 2020). Species from ser. *Rupestria* have some features in common. However, the formation of a triad with a binucleate micropylar cell and plasmodesmata without adjacent electron-dense material, formation of wall ingrowths and presence of plasmodesmata with electron-dense material in the outer walls of antipodal cells are characteristics described only for *S. rupestre* and *S. sediforme*, not for *S. hispanicum*, during megasporogenesis and megagametogenesis*.* Despite the observation of different megasporogenesis events in the three studied species (linear tetrad only in *S. hispanicum*), the embryo sac of *Polygonum* type was noted in all of them. The results reported in the present study and the available embryological data allow concluding that species from ser. *Rupestria* show unique features, not described for the other, previously studied *Sedum* and Crassulaceae representatives, during megasporogenesis, megagametogenesis and embryogenesis. The data collected during the research gave rise to one of the arguments, which supports the statement that this group of plants has a distinct nature. The confirmation of the research hypothesis allows concluding that plasmodesmata with unusual electron-dense material are characteristic of all Crassulaceae species that are ultrastructurally tested so far.

## Conclusion

The studied species *S. rupestre* is characterized by monosporic megasporogenesis and *Polygonum*-type megagametogenesis. Cell wall is not formed between nuclei resulting from meiosis in the micropylar dyad cell; therefore, three cells are formed after megasporogenesis. A single FM is always placed most chalazally in the triad and is mononucleate. Cellularization of the coenocytic megagametophyte leads to the formation of seven cells. Four cell types of the female gametophyte differ morphologically. The cytoplasm of antipodal cells and synergids changes during the development and maturation of the female gametophyte. Three antipodal cells are active, which seem to participate in nutrient transport. The synergids exhibit relatively maximal activity after the degeneration of antipodals, and both synergids can provide nutrients and pollen tube attractants. The presence of electron-dense plasmodesmata was observed in the cell walls of the developing megagametophyte. Plasmodesmata of this type have not been previously described in *S. rupestre*. The observations made so far in the two ultrastructurally tested species from ser. *Rupestria*—*S. rupestre* and *S. sediforme*—are compatible and allow concluding that the lack of plasmodesmata with the electron-dense material during embryogenesis does not exclude the possibility of their formation during megagametogenesis. A comparative analysis of the collected microscopic data showed similarities in cell ultrastructure during megasporogenesis, megagametogenesis and gametophyte in the representatives of ser. *Rupestria*. Embryological results reported in the studies on female gametophyte development of *S. rupestre* can be used as supplementary data for analyzing systematic relationships.
